# Sensitivity of vegetation to climate variability and its implications for malaria risk in Baringo, Kenya

**DOI:** 10.1371/journal.pone.0199357

**Published:** 2018-07-05

**Authors:** Jacinter A. Amadi, Daniel O. Olago, George O. Ong’amo, Silas O. Oriaso, Mark Nanyingi, Isaac K. Nyamongo, Benson B. A. Estambale

**Affiliations:** 1 Institute for Climate Change and Adaptation, University of Nairobi, Nairobi, Kenya; 2 Department of Plant Sciences, Kenyatta University, Nairobi, Kenya; 3 School of Biological Sciences, University of Nairobi, Nairobi, Kenya; 4 Department of Public Health, Pharmacology and Toxicology, University of Nairobi, Nairobi, Kenya; 5 Department of Biomedical Sciences, Colorado State University, Fort Collins, CO, United States of America; 6 Cooperative Development, Research and Innovation, Cooperative University of Kenya, Nairobi, Kenya; 7 Division of Research Innovation and Outreach, Jaramogi Oginga Odinga University of Science and Technology, Bondo, Kenya; Columbia University, UNITED STATES

## Abstract

The global increase in vector borne diseases has been linked to climate change. Seasonal vegetation changes are known to influence disease vector population. However, the relationship is more theoretical than quantitatively defined. There is a growing demand for understanding and prediction of climate sensitive vector borne disease risks especially in regions where meteorological data are lacking. This study aimed at analyzing and quantitatively assessing the seasonal and year-to-year association between climatic factors (rainfall and temperature) and vegetation cover, and its implications for malaria risks in Baringo County, Kenya. Remotely sensed temperature, rainfall, and vegetation data for the period 2004–2015 were used. Poisson regression was used to model the association between malaria cases and climatic and environmental factors for the period 2009–2012, this being the period for which all datasets overlapped. A strong positive relationship was observed between the Normalized Difference Vegetation Index (NDVI) and monthly total precipitation. There was a strong negative relationship between NDVI and minimum temperature. The total monthly rainfall (between 94 -181mm), average monthly minimum temperatures (between 16–21°C) and mean monthly NDVI values lower than 0.35 were significantly associated with malaria incidence rates. Results suggests that a combination of climatic and vegetation greenness thresholds need to be met for malaria incidence to be significantly increased in the county. Planning for malaria control can therefore be enhanced by incorporating these factors in malaria risk mapping.

## Introduction

Despite current gains in malaria reduction, the disease continues to have devastating health and livelihood impacts especially in sub-Saharan Africa where 88% of global malaria cases and deaths occur [1]. In Kenya, malaria accounts for about 18% of outpatient consultations and 6% of hospital admissions [2]. Nearly 75% of the Kenyan population live in malaria prone zones, mostly in epidemic and seasonal transmission zones [3]. Epidemic transmission is influenced by a complex of interacting factors including vector, parasite, climate, environmental and socioeconomic factors [4]. Malaria prevention and control efforts in Kenya are biased towards lake endemic and highland epidemic zones while seasonal transmission zones remain largely neglected [5]. Malaria is among the most prevalent diseases in seasonal transmission zones such as Baringo County [3].

Studies undertaken in East African region reveal trends of changing rainfall and temperature [6,7]. For example, there was a warming of up to 0.6°C in minimum temperature in some parts of East Africa over the period 1939–1992 [6]. Similarly, increasing minimum and maximum temperature trends were observed over the Greater Horn of Africa during the 1961–2010 period [7]. A warming trend is also projected across all seasons and the entire region is anticipated to warm by about 2°C or more by the end of the 21^st^ Century [8]. While models simulate more rainfall for the region in the future, recent instrumental data show a declining trend–this inconsistency is referred to as the “East Africa climate paradox” [9]. For instance, a declining rainfall trends have been observed in parts of Kenya [7]. However, models projected a more than 10% increase in mean precipitation over the semi-arid areas of northern Kenya and a generally wetter climate in the East African region during the mid-21^st^ Century [10]. Earlier, an increase in seasonal and annual precipitation (2–11%) was projected for the region by 2099 [11], though some models showed decreasing rainfall during the long rains season [12].

In many tropical ecosystems, such as savannas, year-to-year variations in vegetation dynamics are controlled primarily by changes in the frequency and timing of precipitation [13]. Vegetation cover offers shade that potentially reduces evaporation, minimizes sub-canopy wind speed, and enhances near ground humidity [14]. Cumulatively, these factors enhance vector population and longevity, and malaria transmissions are likely to increase with increased vector survival [15]. This has been demonstrated in previous studies which showed that mosquito vector populations are likely to be high when vegetation growth is at its peak [16].

Moderate Resolution Imaging Spectroradiometer (MODIS) derived Normalized Difference Vegetation Index (NDVI) has been used to assess, monitor or model land cover changes for various applications ranging from drought early warning systems, health and epidemiology, to biodiversity monitoring and conservation [17–19]. Remote sensing is an indispensable tool for mapping spatial distribution and modelling malaria transmission risks. Climatic and environmental variables are important parameters when modelling malaria transmission [4]. Although studies at various scales have used remote sensing in analyzing spatial and temporal changes in malaria risks, these tools have not been adequately applied in planning for malaria control in remote areas where meteorological data are lacking. This study assessed the seasonal and year-to-year association between climatic factors (rainfall and temperature) and vegetation cover, and its implications for malaria risk in four ecological zones of Baringo County. This study presents, a local scale analysis of climatic and vegetation dynamics using remotely sensed data and further, analyses the relationship with malaria cases in the dryland areas of Northern Kenya. Understanding these dynamics is central to effective control of malaria transmissions and its associated adverse ramifications and for design and implementation of evidence-based risk reduction measures.

## Materials and methods

### Study area

**Fig 1:**
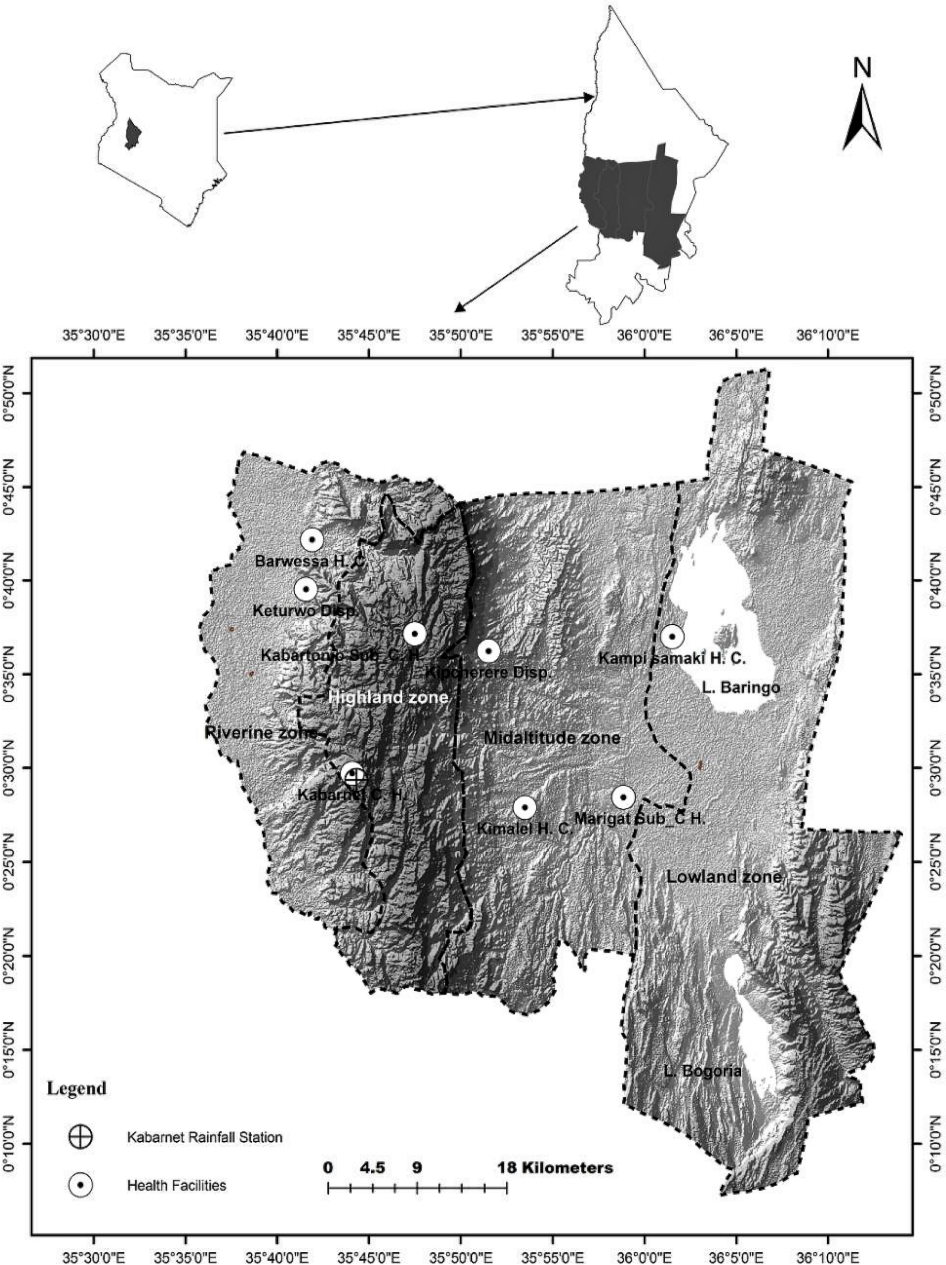
Map of Kenya showing Baringo County and the selected study area.

The mostly arid to semi-arid Baringo County lies between 00° 26' and 00° 32' North and 36° 00' and 36° 09' East. For the purposes of this study, four ecological zones were identified as highland (1500–2300 m a.s.l.), mid-altitude (1000–1500 m a.s.l.), riverine (Kerio valley, 1100–1200 m a.s.l.) and Lake ecosystem (lowland, below 1000 m a.s.l.; Fig 1) based on altitude, vegetation types and climatic characteristics. The mean annual rainfall varies between 300-600mm while mean annual minimum and maximum temperatures are 20°C and 35°C, respectively. The dominant vegetation include the evergreen forest in the highland zone, deciduous bushland and thicket in the mid-altitude zone, woodland and riparian forest in the riverine zone and semi desert grassland and shrub-land in the lowland zone. In the lowland zone, areas around Marigat and Loboi are dominated by invasive *Prosopis juliflora* bushes.

### Climate and environmental data

Land surface temperatures (T_max_ and T_min_) for the period 2004 to 2015 were extracted from the USGS LandDAAC MODIS dataset [20]. Observed climate data were available only for the highland zone thus could not support analysis in other ecological zones. In addition, observed rainfall data were incomplete and not up-to-date. Thus, only observed temperature (monthly minimum and maximum temperatures) data for the highland zone obtained from Kabarnet station for the 2005–2013 period were used to authenticate remotely sensed temperature data. Missing records were filled using the mice package in R software [21]. The Climate Hazards Group InfraRed Precipitation with Station Data (CHIRPS) time-series [22] was used to estimate rainfall in the region over the same time period as for the temperature data. Daily precipitation data with a resolution of 0.05° were used to derive the total monthly and annual precipitation for each zone. Sixteen day mean values of the NDVI was derived from MODIS imagery from the Aqua Satellite for the 2004–2015 period [23]. The 16-day composites were averaged into monthly and seasonal means for each year. All data sets (CHIRPS, MODIS-NDVI and MODIS-land surface temperatures) were downloaded as per the ecological zones. The data were grouped into seasons as follows: (1) “long” rains season—March, April, May (MAM), (2) “short” dry season—June, July, August (JJA), (3) “short” rains season—September, October, November (SON) and (4) “long” dry season—December, January, February (DJF).

### Malaria data

Data on the number of clinical malaria cases were obtained from eight health facilities (Barwessa and Keturwo dispensaries in the riverine zone; Kampi ya Samaki health center and Marigat sub-county hospital in the lowland zone; Kipcherere dispensary and Kimalel health centre in the mid-altitude zone, and Kabarnet and Kabartonjo hospitals in the highland zone) (Fig 1). Patient-level data was not accessed since unique codes were used to replace names in the records. Data on malaria cases was for the 2009–2012 period, this being the only period where records were complete and available for all the named health facilities.

### Data analysis

The Mann-Kendall (M-K) trend test with Sen’s slope estimator was used to assess trends in minimum and maximum temperature, rainfall, and NDVI. Seasonal averages and trends were estimated using Seasonal Mann-Kendall (SMK) for each zone at 5% significance level. Cross correlation function (CCF) was used to determine the relationship between NDVI and lagged (1, 2 and 3-month intervals) climate variables [24]. The temporal records of monthly NDVI were first square root transformed to generate normally distributed data sets [25]. A linear regression model was generated with NDVI as the response variable and rainfall and temperatures as the predictor variables. This method has been employed in other similar studies describing vegetation response to climate factors [19].

Since malaria cases and NDVI data sets were clustered, the continuous data (temperature, rainfall, and NDVI) were discretized into categorical data. A generalized linear mixed model (GLMM) was used with Poisson distribution to model the association between malaria cases, climate (T_min_ and rainfall), and NDVI (Eq 1). The Poisson model allows over-dispersion and is commonly used in environmental epidemiology [26].

$$Ln\left(\gamma_{i}\right)=\ln\left(p\right)+\beta_{1}X_{1}+\beta_{2}X_{2}+\beta_{3}X_{3}+\mu_{i}$$ (1)

Where:

γ_*i*_ = rate of malaria cases at time *i*

p = offset variable; here population size [27]

X = predictors (NDVI, Rainfall, T_min_)

β = coefficients.

$\mu_{i}$ = random effect.

Maximum temperature was excluded from the models due to high correlation with minimum temperature (0.844). Studies have shown that minimum air temperature is primarily linked to malaria transmission due to its influence on vector and parasite development rates [28]. A simple regression model was fit to evaluate the crude effect of each of the predictors on malaria incidence. Then a multiple regression model was fit to evaluate the adjusted effect of the predictors on malaria incidence, as well as, seasonality, differences in ecological zones, and year-to year variations in all variables. In the model, an offset variable was used to standardize the source population to enable comparability of rates. The relative risk of malaria occurrence was estimated using the incidence rate ratio (IRR = cases per unit population) [29]. All statistical analyses were performed in R version 3.2.2 software [30].

## Results

### Annual climatic and vegetation trends

**Fig 2:**
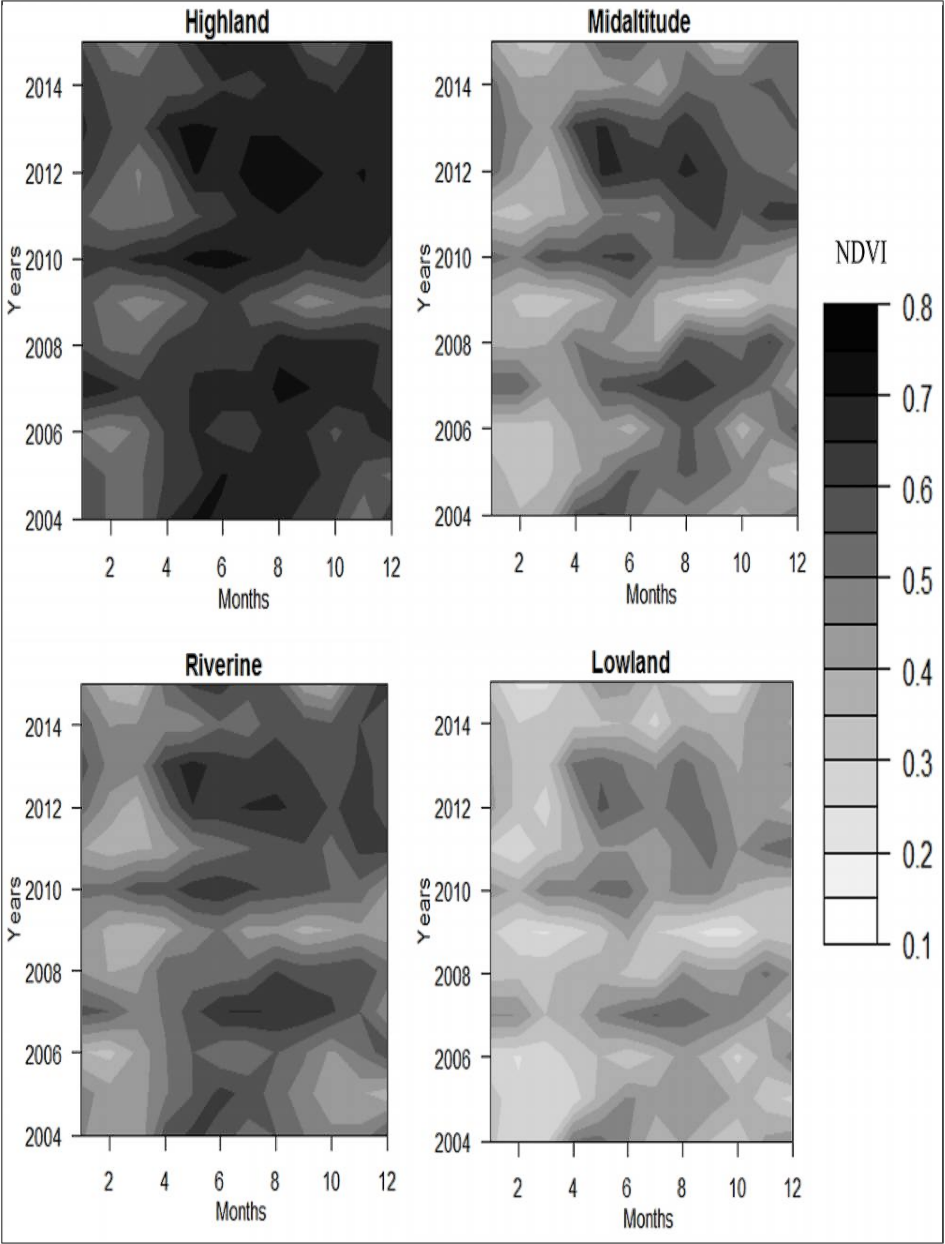
Trend in NDVI change observed during 2004–2015 period. Values are: 1- January, 2—February, 3—March, 4—April, 5 –May, 6 –June, 7 –July, 8, August, 9 –September, 10—October, 11—November, 12 –December.

**Fig 3:**
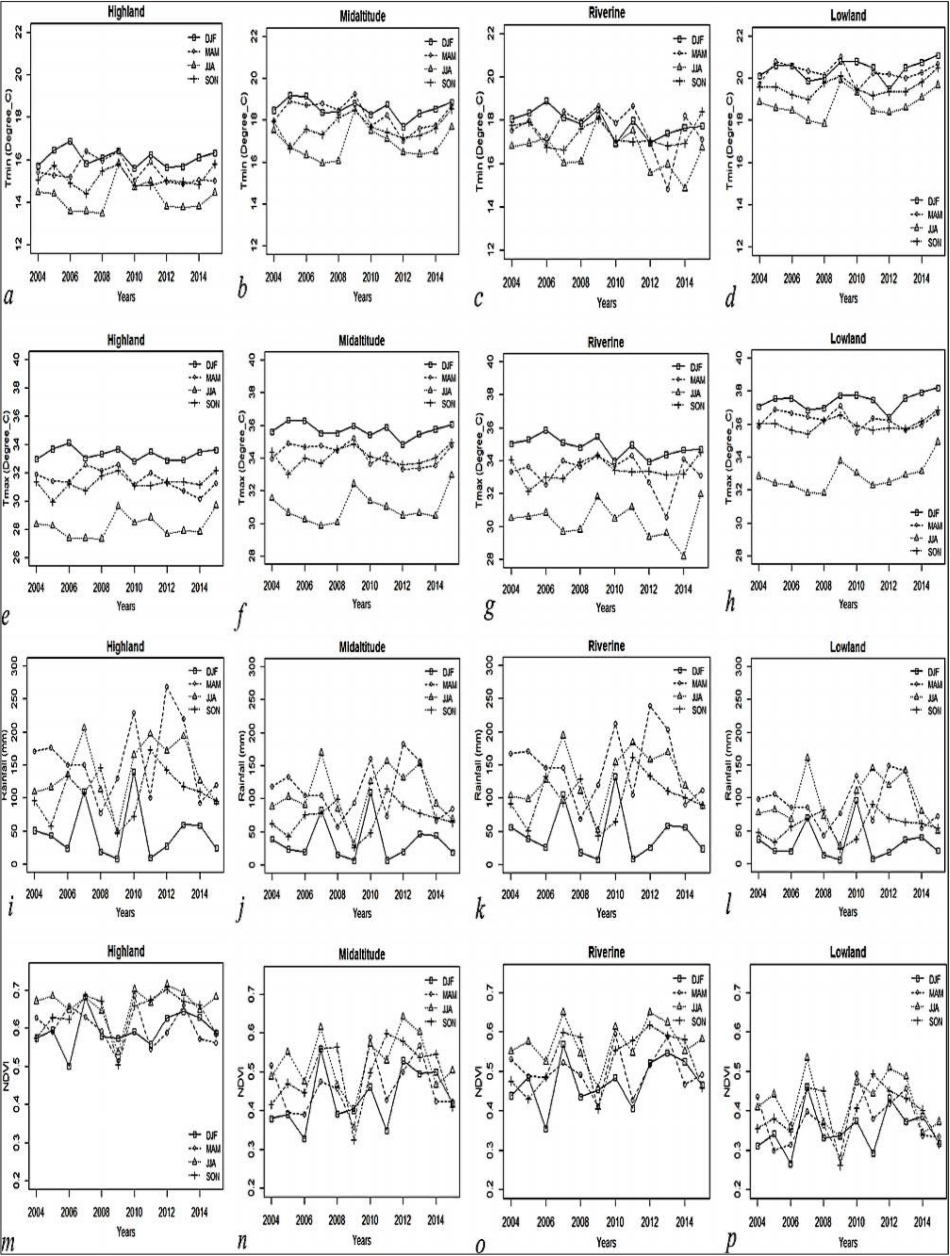
**Seasonal trends in T_min_ (A-D), T_max_ (E-H), rainfall (I-L), and NDVI (M-P)**.

Over the 2004 to 2015 period, the riverine zone had a statistically significant trend for remotely sensed temperatures, marked by a decrease in T_min_ (tau = -0.174; p = 0.0096). In the highland zone, *in situ* temperature trends corroborated remotely sensed temperature data where a significant decrease in mean annual T_min_ was recorded (tau = -0.327; p<0.05). Fig 2 shows the spatio-temporal variation in NDVI observed during the 2004–2015 period. The annual vegetation cover substantially declined in 2009, and regeneration occurred thereafter with peak vegetation greenness observed during 2012–2013 in all zones (Fig 3M–3P). High NDVI was recorded in the highland zone and low NDVI in the lowland zone. During the study period, there was a significant increase in NDVI in the riverine (tau = 0.144; p = 0.011) and mid-altitude (tau = 0.119; p = 0.035) zones that is likely related to enhanced vegetation as a response to the general increase in mean annual precipitation.

### Seasonal trends

There were no change in seasonal trends in mean T_min_ and T_max_ across all zones during the 2004–2015 period. Fig 3A–3H show high seasonal temperatures trends in the lowland zone, DJF season being warmest while JJA the coolest. In contrast, the highland zone received the highest seasonal rainfall and showed maximum NDVI measurements compared to other zones (Fig 3I–3P).

A strong positive relationship was observed between the short rainy season (SON) and the corresponding growth of vegetation. More generally, NDVI values were higher during the SON season compared to the MAM and JJA seasons in the highland and midland zones (Table 1) indicating additive effects of rainfall from the previous seasons.

**Table 1:** Influence of climatic factors on vegetation cover as observed across the four seasons.

	Highland		Mid-altitude		Riverine		Lowland	
	t	*p*	t	*p*	t	*p*	t	*p*
Rainfall	3.720	0.00054*	4.3810	0.000068*	4.295	0.000089*	4.705	0.000024*
T_min_	-1.185	0.242	-1.399	0.169	-2.974	0.00464*	-1.858	0.0696
T_max_	-0.134	0.894	0.403	0.689	1.672	0.101	0.433	0.667
MAM	-2.713	0.00936*	-1.607	0.115	-1.962	0.056	0.716	0.477
JJA	-0.281	0.780	-0.047	0.963	1.069	0.099	-0.298	0.767
DJF	1.020	0.313	0.392	0.697	-0.603	0.550	0.904	0.371

Note: SON is the reference season;

* denotes significance level at 0.05

### Monthly trends

**Fig 4:**
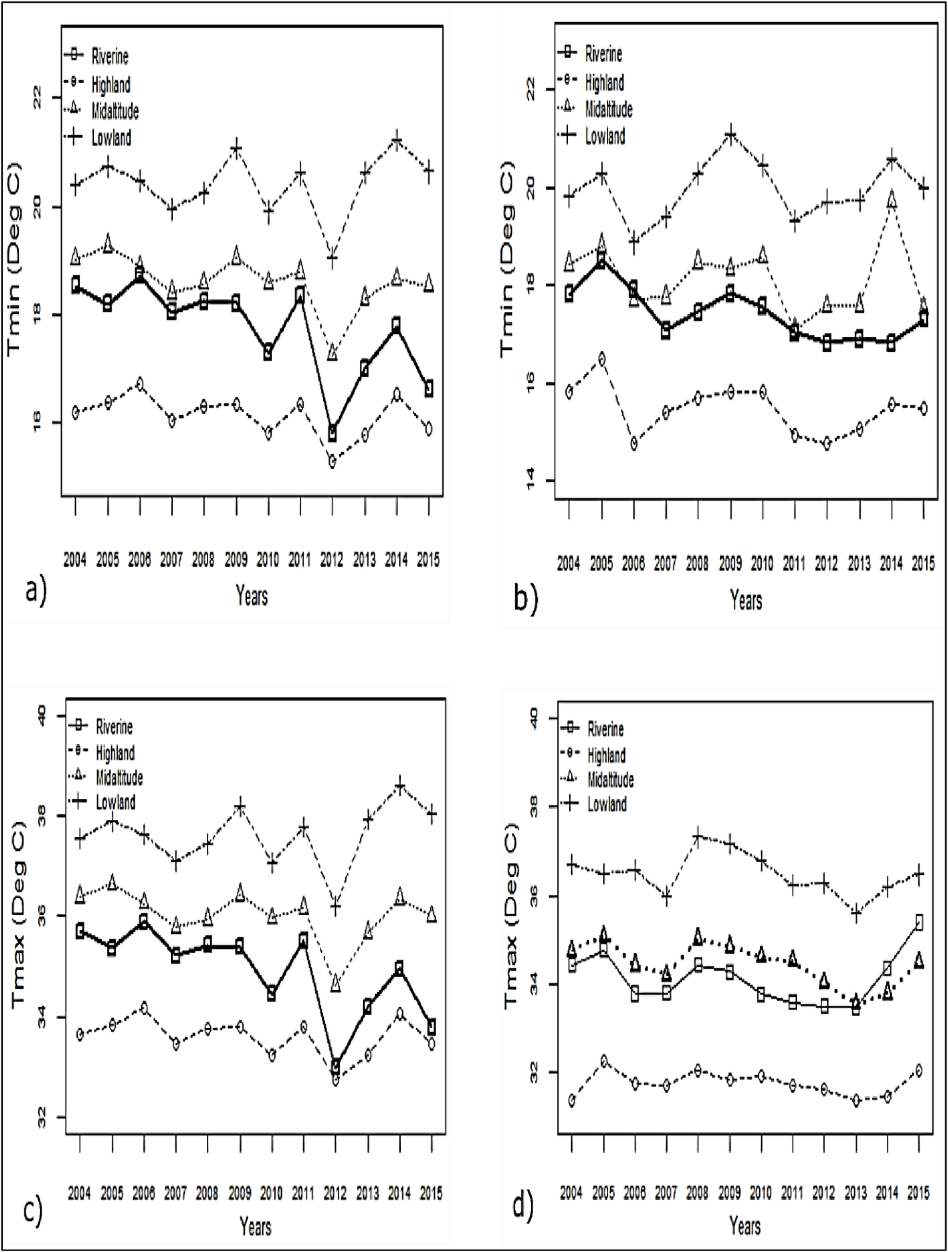
Trends in mean monthly T_min_ and T_max_ for selected months as examples. (A) January, (B) December, (C) January, and (D) October.

The variation in mean T_min_ and T_max_ is shown in Table 2. In the riverine zone, a significant decrease in T_min_ was observed during the months of January and December in the 2004–2015 period (Fig 4A and 4B respectively). Similar significant decreases in T_max_ were noted in the riverine and mid-altitude zones during the months of January and October respectively (Fig 4C and 4D respectively). In contrast, the lowland zone recorded significant increase in T_max_ during July (p = 0.010).

**Table 2:** Monthly minimum and maximum temperature trends in the four zones during 2004–2015.

	Highland		Mid-altitude		Riverine		Lowland	
Month	T_min_	T_max_	T_min_	T_max_	T_min_	T_max_	T_min_	T_max_
	tau	tau	tau	tau	tau	tau	tau	tau
January	-0.273	-0.273	-0.131	-0.217	-0.02*	-0.02*	-0.493	-0.337
February	0.583	0.583	0.784	0.681	0.493	0.583	0.273	0.337
March	0.337	0.1	0.784	0.583	0.411	0.273	0.493	0.891
April	0.217	0.217	0.273	0.337	0.273	0.273	0.891	0.891
May	0.217	0.337	0.337	0.493	0.681	0.891	0.411	-0.273
June	0.217	0.681	0.784	0.493	0.681	0.784	0.784	0.583
July	1	0.493	0.583	0.273	0.891	0.784	0.217	0.01*
August	0.784	0.583	0.583	0.784	0.891	0.681	0.583	0.337
September	0.891	0.217	0.493	0.337	0.584	0.891	0.493	0.17
October	0.981	0.493	0.1	-0.028*	0.273	-0.217	1	-0.17
November	0.583	0.583	0.784	0.784	0.337	0.337	0.681	1
December	0.411	0.891	0.131	0.891	-0.014*	-0.055	0.493	0.131

* denotes significance level at 0.05

**Fig 5:**
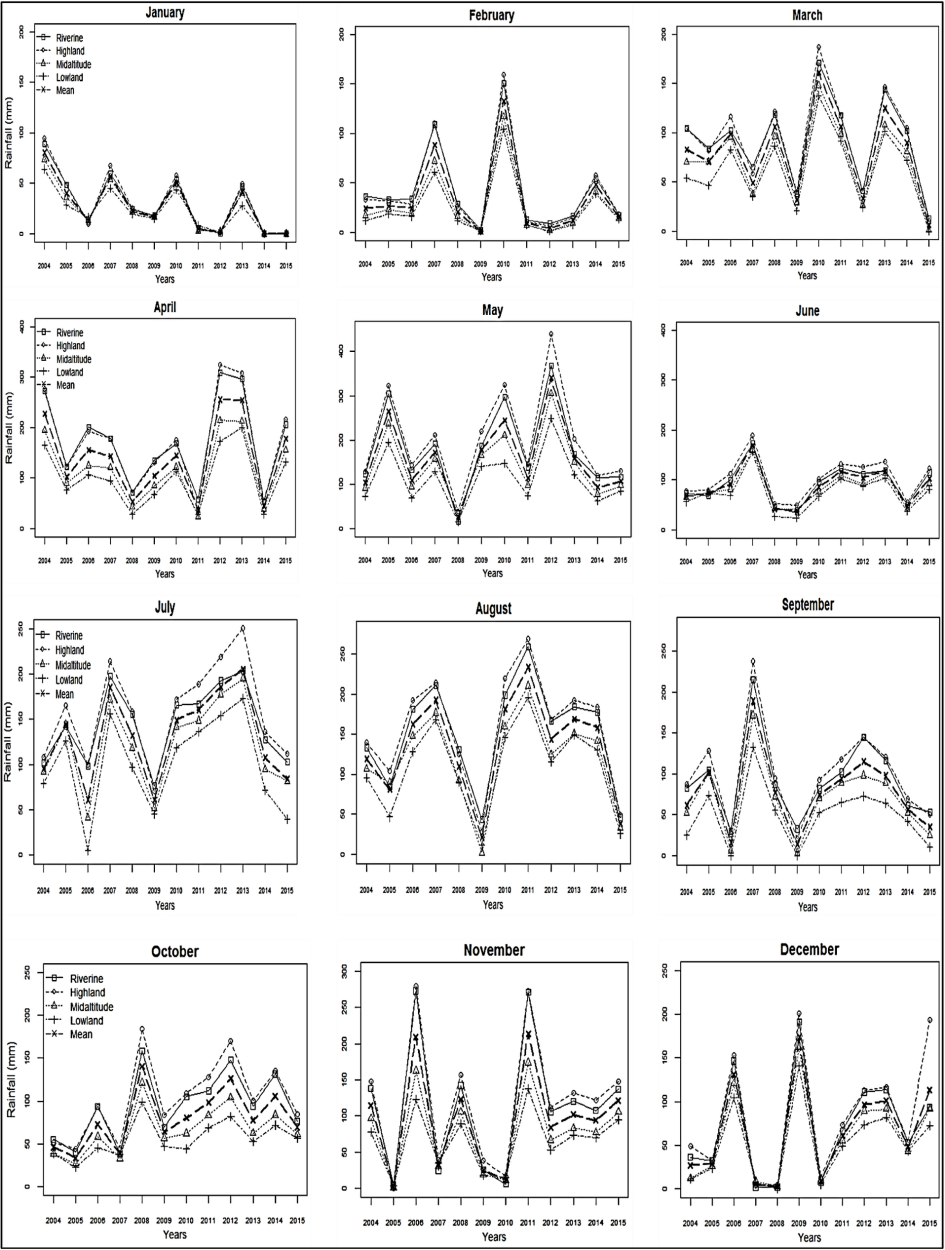
Total monthly rainfall in the four ecological zones during the period 2004 to 2015. A decreasing trend is observed during January while an increasing trend is observed during October.

Trend analysis showed alternate decrease and increase in total monthly rainfall (Fig 5). There was a significant decrease in mean monthly rainfall during January for the 2004–2015 period in the highland (tau = -0.561; p = 0.011), riverine (tau = -0.591; p = 0.007, mid-altitude (tau = -0.561; p = 0.011), and lowland (tau = -0.606; p = 0.006) zones. Similar decreasing trends were observed in March, April, and September, though the decreases were not significant. A significant increase in total monthly rainfall was noted in the lowland zone in October (tau = 0.455; p = 0.040; Fig 5).

**Fig 6:**
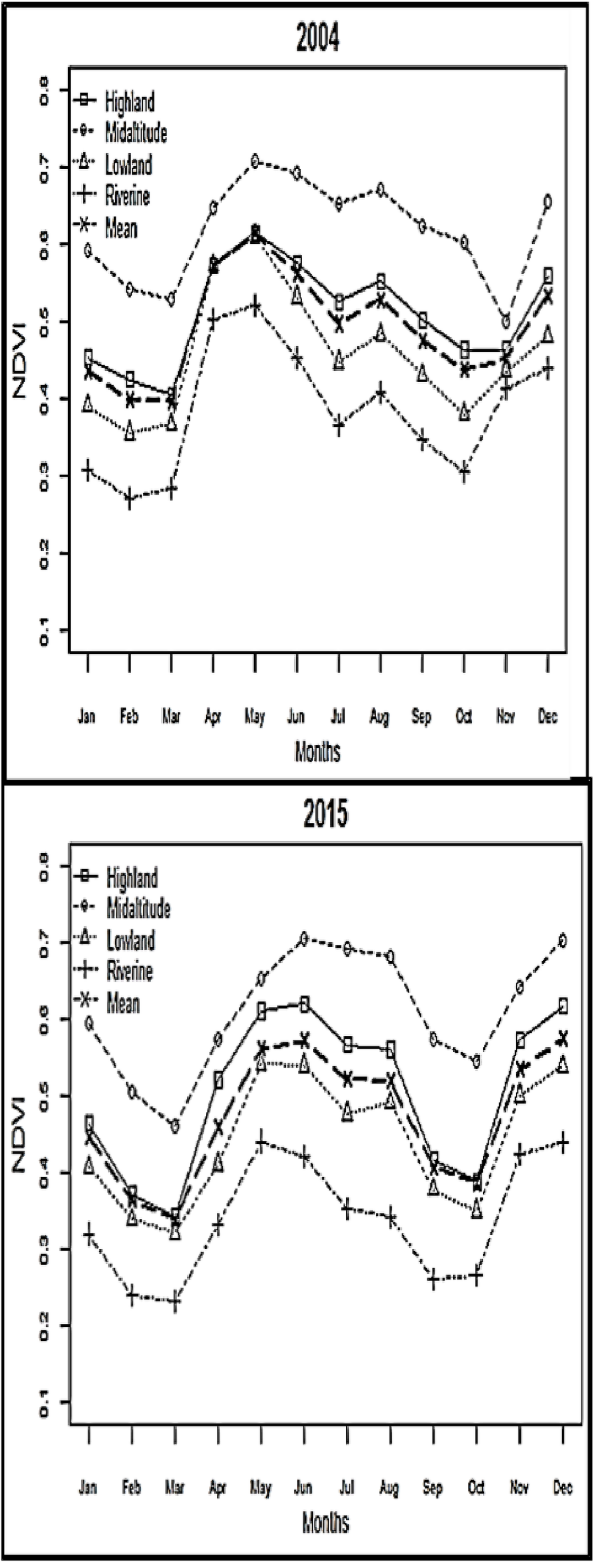
The annual cycle of NDVI for the years 2004 and 2015 as examples. Mean monthly values are indicated in bold.

The annual NDVI decreased between January and March, and steadily increased between April and June (Fig 6). A subsequent decrease was observed between September and October that was followed by an increase between November and December. A significant positive NDVI trend was observed in the riverine zone during November (p = 0.0087) and December (p = 0.033) suggesting that vegetation regeneration in riverine zone could be sensitive to cumulated effects of rainfall from previous months.

NDVI at 1-month lag showed the strongest positive correlation with rainfall. A strong negative relationship was detected between NDVI and temperatures with no time lag for T_min_, and with 1-month time lag for T_max_. In the riverine zone, minimum temperature had a significant negative relationship with NDVI, suggesting that decreasing temperature could have had positive influence on the vegetation growth.

### Malaria risk

**Fig 7:**
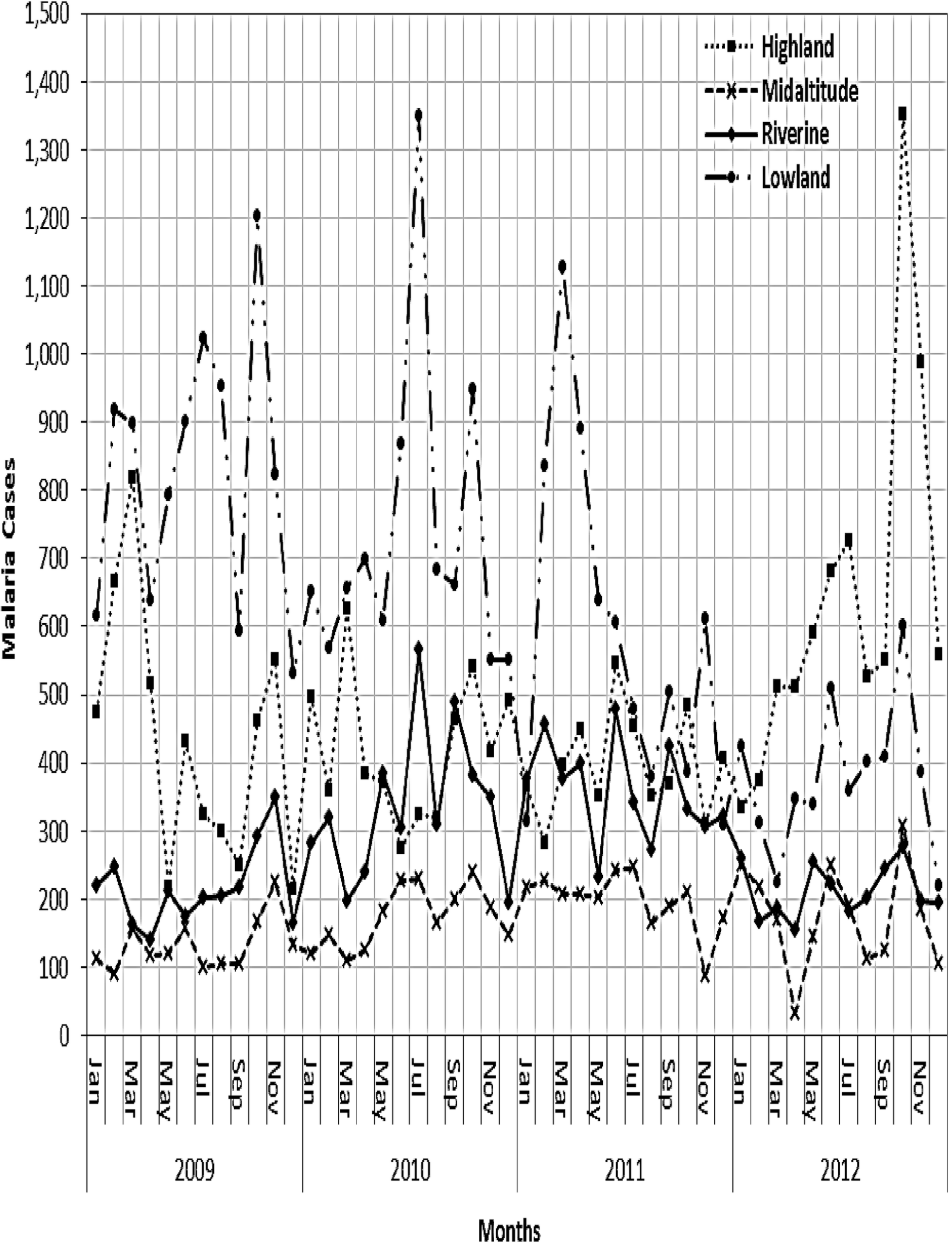
Monthly trends in malaria cases recorded per zone during 2009–2012 period.

**Fig 8:**
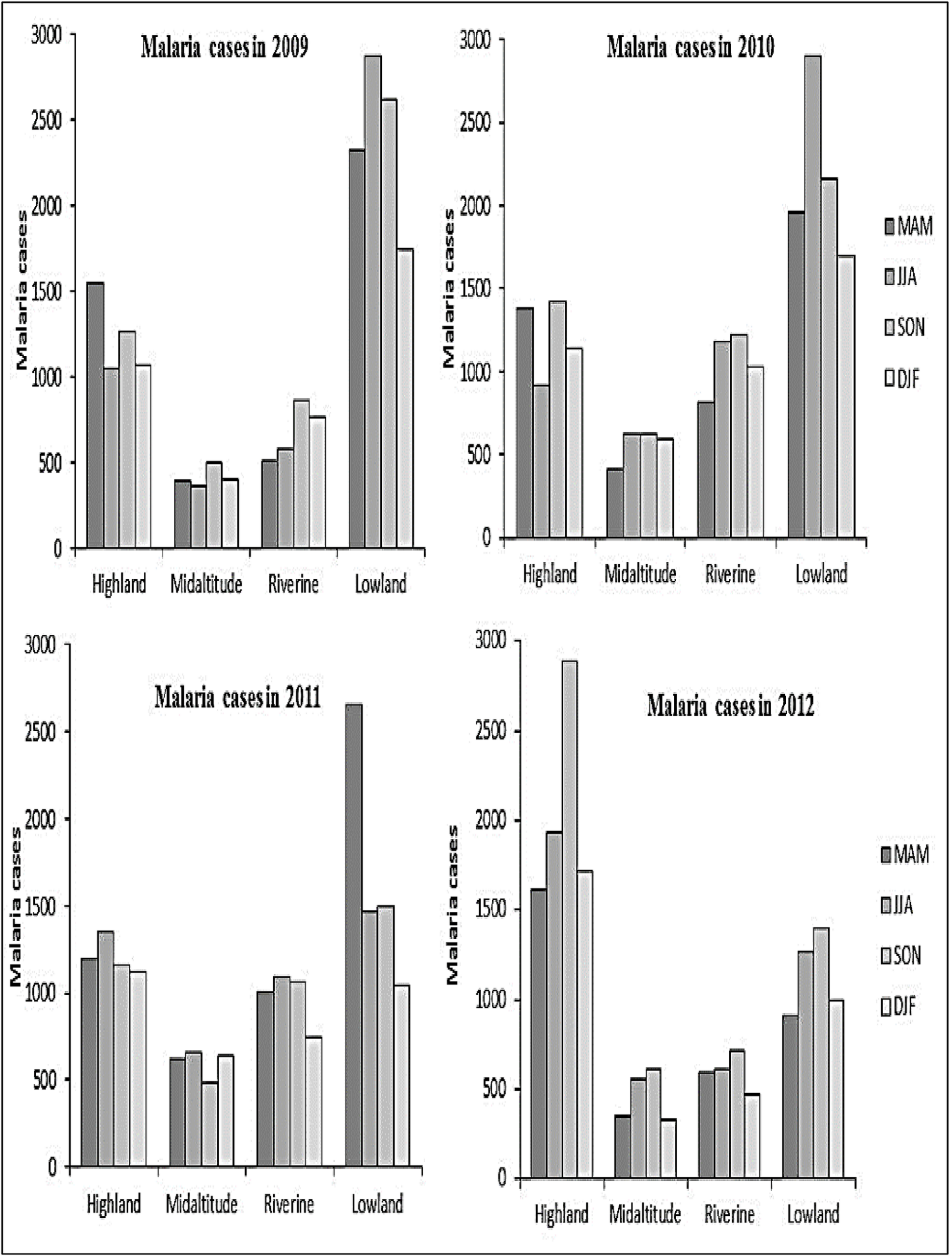
Distribution of total malaria cases by seasons during 2009–2012 period.

Higher malaria cases were observed in the lowland and highland zones during the 2009–2012 period compared to the mid-altitude and riverine zones (Fig 7). Malaria trends showed a seasonal pattern where, in the lowland zone, escalated malaria cases were recorded during the JJA and SON seasons, except in 2011, when higher cases were observed during the MAM season. However, there was no clear seasonal pattern in malaria trends in the riverine and mid-altitude zones (Fig 8).

Reported malaria cases significantly reduced in the lowland zone (tau = -0.583; p<0.01) during the 2009–2012 period. However, a significant increase was observed in the highland (tau = 0.244; p = 0.015) and mid-altitude zones (tau = 0.246; p = 0.013). The decline observed in the lowland zone, is attributed to disparate malaria intervention programs rolled out in the area as opposed to other zones. In the riverine zone, a steady increase in malaria cases occurred during 2009–2011 that was followed by a decline in 2012.

Correlation analysis showed that both minimum temperature and NDVI were significant variables associated with high malaria incidence with a 1-month lag. The correlation between rainfall and malaria cases was highest with a 2-month lag. Malaria cases had significant association with NDVI and climatic factors in the simple regression model (p<0.05). However, on adjusting for the effect of T_min_, rainfall, and seasons, there was a 29.7% increase in malaria risk at T_min_ between 16.2°C—18.6 compared to those below 16.2°C (Table 3). A significant increase in malaria risk was noted at T_min_ above 18.6°C (IRR>1; p<0.05).

**Table 3:** Effect of various ranges of rainfall, T_min_, and NDVI on malaria incidence rate.

Variables	Categories	Range		IRR		*p*
Intercept				0.018		0.0001
	Sparse	0.26–0.41				
NDVI	Moderate	0.42–0.56		0.912		0.0001
	Dense	0.57–0.72		1.076		0.219
	Low	6.50–93.90				
Rainfall (mm)	Moderate	94.00–181.00		1.253		0.0001
	Heavy	181.10–269.00		0.545		0.0001
	Low	13.80–16.20				
T_min_ (°C)	Moderate	16.21–18.60		1.297		0.0001
	High	18.61–21.00		1.685		0.0001
	JJA			1.356		0.001
Season	MAM			1.537		0.0001
	SON			1.591		0.0001

A reduction by 8.8% (p<0.05) in malaria risk occurred when there was moderate vegetation cover compared to sparse vegetation cover (Table 3). Although a 7.6% increase in malaria risk occurred when there was dense vegetation cover (NDVI above 0.57), this risk was not significantly different from that of sparse vegetation cover (NDVI below 0.35).

In this study, monthly rainfall lower than 94 mm had no significant effect on malaria risk while values above 181mm per month caused 45.5% reduction in malaria risk. Thus, a moderate amount of rainfall (between 94-181mm in one month) was necessary for 25.3% increase in malaria risk. Malaria risk was significantly higher during MAM, JJA and SON seasons compared to DJF (IRR>1; p<0.05; Table 3). T_min_ and NDVI accounted for 66% (29.9, 36.1 respectively) of the total variation in malaria incidence explained by model. Differences between the ecological zones and year-to-year variations in vegetation cover, rainfall and minimum temperature had insignificant effects on malaria risk (*p*>0.05).

## Discussion

Time series of monthly, seasonal, and annual patterns of rainfall, minimum and maximum temperature, and NDVI were analyzed at four ecological zones in Baringo County. Significant spatial variations in temperatures were observed. There was a decrease T_min_ trends observed in the riverine and highland zones. Studies have shown that minimum and maximum air temperature trends are neither stable nor increasing but often depict fluctuations over varied temporal scales. While some studies found decreasing T_min_ and T_max_ trends [6,31], warming trends were reported across different parts of Africa [32,33]. The short-term cooling episodes are not unique to this study since similar decreasing minimum temperature trends been previously reported in Serbia [34] and Northeast Brazil [35] during 2000–2010 and 1960–2011 respectively. Suggested reasons for such cooling trends include the influence of large water body causing alterations in meso-scale circulations [6,35], and decadal cooling of the Tropical Pacific sea surface temperature (SST) [36]. The observed warming trends in the lowland zone corresponds with studies in different locations in East Africa [6,32]. Although the recent global warming has been largely attributed to anthropogenic effects [37], the reasons for the observed local-scale temperature slowdown are indistinctive and merits further inquiry.

The observed rainfall during January and MAM season in all zones corroborate other studies over Kenya and other parts of East Africa [38]. While observations and simulated ensembles suggest that decreasing long rains over the East African region is linked to the SST anomalies [39] the increasing short rains has been attributed to the warming of the western Indian Ocean [40]. The decreased rainfall observed during the MAM season is primarily related to declines during the months of March and April whereas the observed increase in the SON season is mainly due to increasing rainfall during the month of October.

This study shows that peak vegetation greenness occurred during the months of November and December, coinciding with the increased short rains. The decreased vegetation cover observed in 2009 is linked to the 2008–2009 drought experienced in the region. The altitudinal variation in overall and annual vegetation greenness is an indication of the differences in response among the vegetation types (mainly forest, woodlands, and shrubs) to rainfall effects. For instance, the presence of evergreen *P*. *juliflora* bushes in the lowland zone may have contributed to the non-significant changes in vegetation in the area. The persistent greenness observed in the highland zone could be linked to dominant forest cover unique to this zone. However, the observed increase in vegetation cover in the riverine and mid-altitude zones may be due to the emergence of open deciduous shrubs that are highly responsive to slight increases in rainfall. The observed annual increase in vegetation trends in all zones in Baringo contradicts studies over Eastern and Central parts of Kenya that showed declining vegetation density during 2001–2010 period [41]. The declining vegetation productivity over Eastern and Central Kenya were attributed to significant decreases in annual rainfall [38]. In the semi-arid tropics, high annual NDVI measurements often corresponds with high annual precipitation [42]. Consequently, increasing vegetation greenness observed in Baringo could be due to the dominant vegetation (woodland to bushland) response to increasing annual rainfall. A study in East Africa showed that woody vegetation growth is a function of accumulated rainfall over multiple months [43].

A positive relationship with one-month lag was observed between rainfall and vegetation-NDVI in all zones. This suggests that vegetation growth response is controlled by the preceding months’ rainfall. This study findings corresponds to those that found up to one-month lag of peak vegetation to rainfall in western Kenya [44]. A non-significant relationship between vegetation greenness and precipitation was reported in Mongolia, China [19], suggesting differences in vegetation response in different geographic regions. Another study revealed that vegetation growth over Sudanian region depended on the history of rainfall in that area [42]. Like drought, the rainfall lag effects on vegetation growth vary from months to years [42].

A negative response of vegetation to temperature occurred with up to 1-month lag. T_min_ at current time (lag 0) had significant negative association with vegetation in the riverine zone. This may be attributed to the strong dependence of woody vegetation in the riverine zone to soil moisture. The observed decrease in minimum temperature in the riverine zone may have caused a reduction in evaporative demand consequently increasing soil moisture available for vegetation growth. Further, the negative temperature-vegetation association could be the result of positive precipitation effects on vegetation that cools the atmospheric air through evaporation [45].

Mean monthly minimum temperature was positively associated with monthly malaria incidence. Moderate to high minimum temperatures (16.2°C—21.0°C) were associated with high malaria incidence rates likely due to increased parasite and mosquito development rates that together enhance malaria transmission [46]. This temperature range was well within the range suggested by studies in Ethiopian highlands [18] and Western Kenya [47] where greatest malaria transmission was reported to occur at temperatures between 17°C and 21°C. Elsewhere, studies have shown that maximum temperature is less significant in malaria transmission [28] and risk prediction [48] compared with minimum temperature. A recent model that incorporated laboratory–based data indicated that temperatures from 16 to 34°C are a potential temperature range for malaria transmission [49]. Certainly, mosquito growth and plasmodium development rate reduce significantly at temperatures lower than 16°C [47,50]. In addition to tolerating higher temperatures (32°C), densities of *An arabiensis* (the dominant malaria vector in Baringo) significantly positively correlated with minimum temperature in Kenya [51].

The high malaria risk observed when total monthly rainfall was in the moderate category (Table 3: 94 -181mm) suggests optimal rainfall levels necessary for the occurrence of malaria outbreak. Monthly rainfall amounts higher than 181mm was associated with decreased malaria incidence, indicating the indirect negative effect of high rainfall on mosquito survival [52]. Conversely, in Burkina Faso a significant increase in malaria incidence was reported when total monthly rainfall was above 100 mm while levels below 90 mm did not have any effect on malaria [50]. In the highlands of western Kenya, a threshold of 80mm to 130mm for total monthly rainfall was associated with significant increase in malaria admissions [53]. Rainfall has both direct and indirect impact on malaria outbreaks, moderate amounts of rainfall influence larval population though its effect on availability of breeding sites [50]. However, excessive rainfall flushes out larvae from habitats reducing mosquito densities consequently lowering malaria risk [52].

In the model, high malaria cases occurred at NDVI values below 0.35. These results were agreeably within the range reported for increased malaria mortality in Western Kenya (0.3–0.4) [44] and for increased malaria cases in Bangladesh [54]. However, a research in the highlands of Ethiopia reported lack of association between vegetation cover and malaria incidence [18]. Lower NDVI values may indicate the onset of vegetation greening and an indicator of surface water availability and near surface humidity [18]; factors that promote vector survival. Further, studies have shown that mosquito vectors are attracted to particular vegetation types due to variations in floral sugar content. For example, a study conducted in Mali found that invasive *Prosopis juliflora* (dominating the lowland zone in Baringo) bushes supported high *Anopheles gambiae s*.*l*. abundance thus increasing malaria transmission risks [55].

Mean monthly minimum temperature and NDVI indicated 1-month lagged effect on malaria cases. These results were in agreement with studies in Anhui China [56]. Rainfall indicated a two months lagged effects on malaria cases corresponding with studies that have reported between 1–3 months rainfall lagged effects on malaria incidence [57]. Such lags are related to the duration needed for mosquito growth and sporozoite development and the specific environmental settings.

## Conclusions

A significant decrease in minimum temperatures was observed in the riverine zone while a general warming trend was observed in the lowland zone, with a significant increase noted during the month of July. A substantial increase in vegetation greenness linked to precipitation occurred in the riverine and mid-altitude zones with 1-month lag. Further, the study confirms that remotely sensed NDVI, rainfall, and minimum temperature are suitable indicators for malaria risk prediction. Future climatic changes will likely alter these environmental conditions increasing malaria risk. Therefore, these factors should be considered when planning for malaria control and risk mapping.
